# Unweaving the role of nuclear Lamins in neural circuit integrity

**DOI:** 10.15698/cst2018.09.151

**Published:** 2018-09-10

**Authors:** Samantha L. Deal, Shinya Yamamoto

**Affiliations:** 1 Program in Developmental Biology, Baylor College of Medicine (BCM), Houston, TX 77030.; 2 Department of Molecular and Human Genetics, BCM, Houston, TX 77030.; 3 Department of Neuroscience, BCM, Houston, TX 77030.; 4 Jan and Dan Duncan Neurological Research Institute, Texas Children’s Hospital, Houston, TX.

**Keywords:** Lamin, neurodegeneration, dopaminergic neuron, giant fiber circuit, Drosophila

## Abstract

Lamins are type-V intermediate filament proteins that comprise the nuclear lamina. Although once considered static structural components that provide physical support to the inner nuclear envelope, recent studies are revealing additional functional and regulatory roles for Lamins in chromatin organization, gene regulation, DNA repair, cell division and signal transduction. In this issue of *Cell Stress*, Oyston *et al*. (2018) reports the function of *Lamin* in the maintenance of nervous system integrity and neural circuit function using *Drosophila*. A number of laminopathies in humans exhibit age-dependent neurological phenotypes, but understanding how defects in specific neural cell types or circuitries contribute to patient phenotypes is very challenging. *Drosophila* provides a simple yet sophisticated system to begin untangling the vulnerability of diverse neuronal cell types and circuits against cellular stressors induced by defects in nuclear lamina organization.

Structural integrity of the nuclear membrane is essential for the homeostasis of eukaryotic cells. Critical components of this process are Lamins or type-V intermediate filament proteins that form a meshwork on the inner nuclear membrane 1. In fact, mutations in genes that encode *Lamins* and related proteins cause a class of diseases referred to as ‘laminopathies’. In the human genome, there are three genes (*LMNA*, *LMNB1*, *LMNB2*) that encode four major Lamin proteins, and biochemists have classified these into A-type and B-type based on their structural features and biochemical properties 2. Two A-type Lamins, Lamin A and Lamin C, are both made from the *LMNA* gene through alternative splicing, whereas two B-type Lamins, Lamin B1 and Lamin B2, are transcribed from *LMNB1* and *LMNB2* genes, respectively. One major difference between these two types of Lamins is that type-B Lamins undergo farnesylation at their C-terminal CaaX box that regulates their intranuclear localization, while mature type-A Lamins lack this modification. The two types of Lamins also differ in their expression patterns. While type-B Lamins are ubiquitously expressed throughout development, type-A Lamins are expressed at later stages when cells undergo differentiation 3. Although most post-differentiated cells express both types of Lamins, some cell types, such as naïve T and B cells of the immune system, lack type-A Lamin expression 4. Furthermore, type-A and type-B Lamins are not fully redundant; each type of Lamin forms their own intermediate filament networks and have distinct functions in the nucleus 5.

Based on OMIM (Online Mendelian Inheritance in Man, https://www.omim.org/) 6, there are 15 types of laminopathies reported in humans that are caused by mutations in the three genes that encode Lamin proteins (**Table 1**). Mutations in genes that encode proteins that post-translationally modify Lamins (e.g. *ZMPSTE24*) as well as proteins that physically interact and function with them (e.g. *EMD*, *LBR*) can cause other forms of laminopathies. Laminopathies that are associated with the *LMNA* gene are grouped as primary laminopathies, and more than 600 different mutations have been reported to date 7. Different mutations in *LMNA* can result in very different phenotypes, including muscular dystrophy, cardiomyopathy, axonal neuropathy, and progeria 8. Although some patterns have emerged through functional studies of disease-associated mutations (e.g. progeria phenotype is caused by a gain-of-toxic function of aberrant LMNA), a clear-cut genotype-phenotype correlation is yet to be established, adding another layer of complexity in understanding the molecular mechanisms that underlie these disorders. Many laminopathy studies have focused on aging-related symptoms due to the striking premature aging phenotypes observed in Hutchinson-Gilford progeria (MIM #176670) 9. In addition, many investigators have been interested in the function of Lamins in cardiac and skeletal muscles since most of the laminopathies exhibit striking symptoms in these tissues 10. In contrast, the function of Lamins in the nervous system, particularly in the aging brain, is still understudied 11. Conventional as well as conditional gene knockout studies in mice revealed that loss of *LmnB1* or *LmnB2* led to neurodevelopmental phenotypes 12,13. Furthermore, double knockout mice for both *LmnB1 *and *LmnB2 *exhibited severe brain atrophy and microcephaly 13,14, indicating that type-B Lamins are necessary for neuronal survival. However, it is unclear whether a milder reduction of Lamin levels in the neurons causes neurodegenerative and/or functional consequences. Such experiments would not only enhance our understanding of laminopathies but may also provide insights into more common neurodegenerative diseases such as Alzheimer’s disease, a condition in which reduction of type-B Lamins and subsequent alterations in neuronal nuclear architecture have been implicated in its pathogenesis 15,16.

**Table 1:** TABLE 1. List of human laminopathies that are caused by mutations in *Lamin* genes. Whether the patient of these diseases have been reported to exhibit neurological symptoms have been extracted from OMIM [https://www.omim.org/(Accessed on 8/31/2018)]. Abbreviations: AR (Autosomal Recessive), AD (Autosomal Dominant), CNS (Central Nervous System), PNS (Peripheral Nervous System).

**Gene**	**Disease Name(Abbreviation or Alternative Nomenclature if Any)**	**MIM #**	**Modes of Inheritance**	**Neurological Presentation**
***LMNA***	Dilated Cardiomyopathy 1A (CMD1A)	115200	AD	No
	Familial Partial Lipodystrophy type 2 (FPLD2)	151660	AD	Yes (PNS)
	Autosomal Dominant Limb-Girdle Muscular Dystrophy type 1B (LGMD1B)	159001	AD	No
	Hutchinson-Gilford Progeria (HGPS)	176670	AD, AR	No
	Autosomal Dominant Emery-Dreifuss Muscular Dystrophy 2 (EDMD2)	181350	AD	No
	Dilated Cardiomyopathy and Hypergonadotropic Hypogonadism (Malouf syndrome)	212112	AD	Some patients (CNS)
	Mandibuloacral Dysplasia type A with Partial Lipodystrophy (MADA)	248370	AR	No
	Lethal Restrictive Dermopathy	275210	AR	No
	Charcot-Marie-Tooth type 2B1 (CMT2B1)	605588	AR	Yes (PNS)
	Slovenian type Heart-Hand Syndrome	610140	AD	1 patient (PNS)
	LMNA-related Congenital Muscular Dystrophy (MDC)	613205	AD	Yes (CNS)
	Autosomal Recessive Emery-Dreifuss Muscular Dystrophy 3 (EDMD3)	616516	AR	Yes (PNS)
***LMNB1***	Autosomal Dominant Adult-onset Demyelinating Leukodystrophy (ADLD)	169500	AD	Yes (CNS)
***LMNB2***	Susceptibility to Acquired Partial Lipodystrophy (APLD)	608709	AD	No
	Progressive Myoclonic Epilepsy 9 (EPM9)	616540	AR	Yes (CNS)

In addition to vertebrate model systems such as zebrafish, mice and human derived tissues and cells, invertebrate model organisms such as *Caenorhabditis elegans* 17,18 and *Drosophila melanogaster* 19,20 have contributed to understanding the *in vivo* function of Lamins. Based on phylogenetic studies, the last common ancestor of vertebrates and invertebrates (urbilaterian) likely possessed a single *Lamin* gene that encodes a type-B Lamin protein 21. *C. elegans* and most invertebrate species have only one *Lamin* gene (*lmn-1* gene in *C. elegans*), whereas the *D. melanogaster* genome carries two genes. Although these genes are thought to have arisen independently from vertebrate *Lamin* genes, one gene,* Lamin C* (*LamC*), shows features of a type-A Lamin whereas the other gene, *Lamin* (*Lam*, also referred to as *Lamin Dm*) has characteristics of a type-B Lamin 22,23. For example, *Lam* is expressed ubiquitously and the encoded protein undergoes farnesylation, whereas *LamC* expression is developmentally regulated and the protein lacks a CaaX motif. Interestingly, *LamC* is not expressed in the nervous system 24,25, simplifying the functional study of Lamins in the brain of *Drosophila*.

In this issue of *Cell Stress*, Oyston, Neely and colleagues assessed the functional consequences of reduction of Lamin levels in the nervous system by performing neuronal specific knockdown of *Lam* in *Drosophila *26. Previous studies in flies have reported shortened lifespan and locomotion defects accompanied by histological signs of neurodegeneration in the brains of hypomorphic *Lam* mutants 16,25,27. However, these studies did not examine whether these effects were due to cell-autonomous defects in neurons or due to systemic effects. By combining histological examinations, behavioral assays and electrophysiological measurements *in vivo*, the authors found that reduction of *Lam* specifically in post-differentiated neurons causes short lifespan and age-dependent motor deficits, accompanied by loss of subsets of neurons and alterations in specific neural circuit function (**Figure 1**). Using two independent UAS-RNAi lines against *Lam* and a neuronal specific GAL4 driver (*elav^C155^-GAL4*), which effectively knockdown mRNA and protein levels, Oyston and colleagues identified that flies with reduced neuronal *Lam* exhibited gradual loss of motor function as measured through a climbing (negative geotaxis) assay. Interestingly, the authors did not observe overt changes in brain morphology that correlated with the motor deficit and shorted lifespan in *Lam* knockdown flies. Since a previous study reported similar age-dependent motor deterioration upon loss of dopaminergic neurons in the PAM (Protocerebral Anterior Medial) cluster 28, the authors performed immunohistochemical assessment of dopaminergic neurons in the brain. Interestingly, while they did not observe any histological defects in most dopaminergic clusters of the adult brain, they found significant decrease in the number of PAM cluster neurons (**Figure 1**), consistent with their behavioral data.

**Figure 1:**
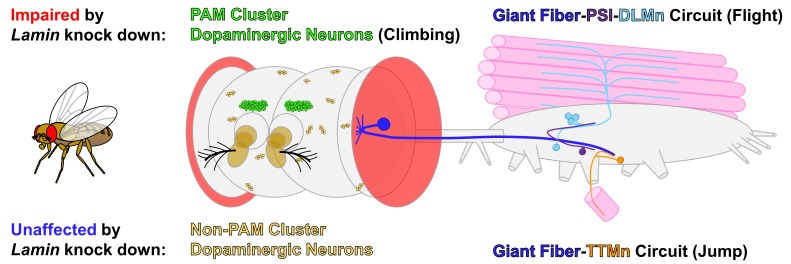
FIGURE 1: Specific neurons and neural circuits in *Drosophila* are more sensitive to reduction of Lamin levels. Schematic diagram of the adult fly central nervous system (brain and thoracicoabdominal ganglion) highlighting the neurons and neural circuits that were studied in Oyston *et al*. 2018. Eyes, antennae and certain muscles are also depicted as landmarks. For simplicity, only one side of the bilateral giant fiber system is shown here. Flies with decreased neuronal *Lamin* expression exhibited age-dependent motor deficits and shortened life span. The authors observed histological or functional defects in PAM (protocerebral anterior medial) cluster dopaminergic neurons and in escape response mediating neuronal circuits composed of giant fiber neurons, peripherally synapsing interneurons (PSI) and dorsal longitudinal motor neurons (DLMn). In contrast, non-PAM cluster dopaminergic neurons and a parallel giant fiber circuit comprised of tergo-trochanteral motor neurons (TTMn) were not affected.

To further assess whether the reduction of *Lam* in the nervous system alters neuron function, the authors turned to the giant fiber system, a well-documented neural circuit that is accessible for *in vivo* electrophysiological recordings in adult flies 29,30. The giant fiber system is composed of two parallel circuits that control two groups of muscles that are essential for a rapid escape response (**Figure 1**). Firing of giant fibers, also known as giant descending neurons, induced through a specific visual stimulus (looming object), triggers simultaneous activation of the two circuits. In the first circuit, giant fibers synapse directly onto motor neurons (TTMn) that regulates the contraction of the tergotrochanteral muscle (TTM), also known as the ‘jump muscle’. The giant fiber-TTMn synapses are primarily mediated by electrical gap junctions (electrical synapse), allowing fast transmission of action potentials. In the second circuit, giant fibers synapse onto peripherally synapsing interneurons (PSI), which in turn synapse onto motor neurons (DLMn) that regulate dorsal longitudinal muscles (DLM), also referred to as the ‘indirect flight muscles’, that are critical for flight. Since the PSI-DLMn connection is predominantly composed of chemical synapses, the response time to trigger the ‘flight program’ is slower compared to the TTMn-mediated ‘jump response’. Hence, the firing of the giant fiber system allows immediate triggering of a quick jump action by the TTM upon identification of an object that is quickly approaching (e.g. a fly swatter), followed by activation of a coordinated flight program through the DLM to fly away from a potential threat. Oyston *et al*. identified that while* Lam* reduction led to age-dependent failure in the DLM branch of the giant fiber circuit, interestingly, they did not observe any significant defects in the TTM branch, suggesting that some circuits are more sensitive to reduction of *Lam* levels than others.

The finding that certain neuronal cell types (e.g. PAM cluster dopaminergic neurons) and circuits (e.g. giant fiber-PSI-DLM circuit) in the fly nervous system are more sensitive to the reduction of *Lam* provide a novel basis for understanding the role of Lamins in the nervous system. It is possible that the differences in the vulnerability may be arising due to the differences in endogenous expression levels of *Lam* in different cell types or through differences in the knockdown efficiency of *Lam *via RNAi (e.g. difference in the levels of GAL4 expression, efficiency of dsRNA processing), which needs to be further tested using different GAL4 drivers and other genetic tools. However, it is interesting to hypothesize that *Lam* may play different roles in different neuronal cell types to maintain the cells’ function and health. In mice, the complete loss of type-B lamins in cone photoreceptors (*^HRGP-Cre^ Lmnb1^Δ/Δ^Lmnb2^Δ/Δ^*) do not cause any major morphological or functional defects 31, while a similar manipulation in the forebrain (*^Emx1-Cre^ Lmnb1^Δ/Δ^Lmnb2^Δ/Δ^*) leads to atrophy of the cortex and hippocampus 13. Hence, the concept of different neuronal populations having different vulnerabilities to reduction of Lamin levels may be evolutionarily conserved. In their manuscript, Oyston *et al*. propose that neural circuits that depend on chemical synapses may be more sensitive to Lamin loss based on the difference they observed between DLM and TTM. While this is an interesting theory, additional electrophysiological studies in other neural circuits that require electrical and chemical synapses will be critical to determining whether this observation can be generalized beyond the giant fiber system. In the manuscript, the authors also propose that the defect they observe in the DLM pathway may be due to loss of neuromuscular junction integrity, or through trans-synaptic effects that alter the responsiveness of the post-synaptic muscle. Future studies investigating the histological and ultrastructural features that are caused by the reduction of *Lam* in the giant fiber system will likely clarify the mechanism that underlies the age-dependent functional deterioration of the DLM branch of the giant fiber system. In the *Xenopus* visual system, mRNA for Lamin B2 is transported to axons of retinal ganglion cells for local translation 32. In this context, Lamin B2 is translated in distal axons and localizes to the mitochondria where it supports axonal integrity by maintaining proper mitochondrial membrane potential and morphology, as well as axonal transport of lysosomes. Further investigation of the *Drosophila* giant fiber system focusing on the potential extra-nuclear function of *Lam* in axonal integrity may provide additional molecular insights into the role of Lamins in neural circuit maintenance.

In summary, the data presented by Oyston *et al.* revealed that neuronal specific reduction of *Lam* recapitulates the neurodegenerative phenotype seen in hypomorphic *Lam* mutants. Their finding that some cells and neural circuits are more vulnerable to the reduction of *Lam* levels provides important insights into our understanding of age-dependent neuronal phenotypes observed in a subset of laminopathies in humans. For example, two individuals from a consanguineous family that presented with progressive myoclonic epilepsy 9 (MIM #616540) were found to be homozygous for a functional missense mutation (p.H157Y) in *LMNB2* 33. Epilepsy in these patients began when they were 6 or 7 years old, respectively, and progressed over time. While these phenotypes may be due to developmental defects in neuronal migration as reported in *Lmnb2* knockout mice, differences in vulnerability for *LMNB2* loss-of-function in post-differentiated neurons may also contribute to phenotypes that appear and progress in an age-dependent manner. In addition, duplications 34 and regulatory mutations that increase the expression of *LMNB1* 35 cause adult-onset autosomal dominant leukodystrophy (MIM #169500), a slow progressive neurodegenerative disease. Future complementary studies in *Drosophila *could assess whether certain cells and circuits are more vulnerable to increases in the level of type-B Lamins and thus may provide insights into why certain neuronal functions are affected more than others in this disorder.
